# Sediment Remediation with New Composite Sorbent Amendments to Sequester Phosphorus, Organic Contaminants, and Metals

**DOI:** 10.1021/acs.est.1c02308

**Published:** 2021-08-26

**Authors:** Johan Wikström, Stefano Bonaglia, Robert Rämö, Gunno Renman, Jakob Walve, Johanna Hedberg, Jonas S. Gunnarsson

**Affiliations:** † Department of Ecology, Environment and Plant Sciences (DEEP), Stockholm University, 106 91 Stockholm, Sweden; ‡ Department of Sustainable Development, Environmental Sciences and Technology, Division of Water and Environmental Engineering, KTH Royal Institute of Technology, 100 44 Stockholm, Sweden

**Keywords:** in situ sorbent amendment, thin-layer capping, eutrophication, metal contamination, HOC contamination, biogeochemical cycles, methanogenesis

## Abstract

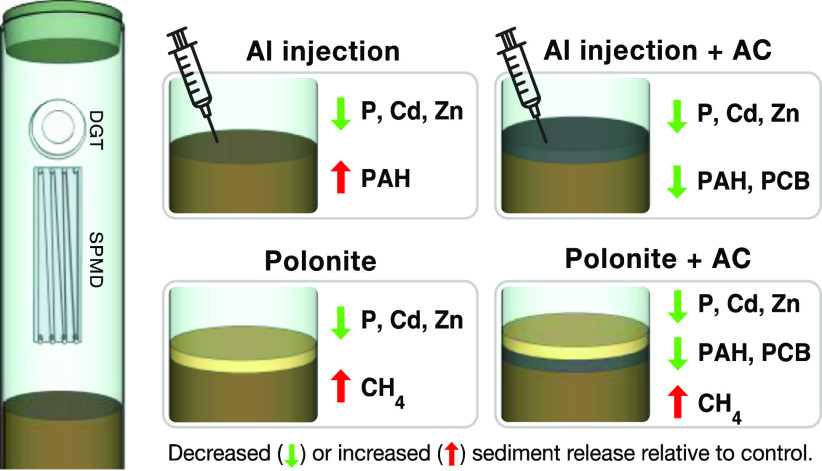

This study tested two sediment amendments with active sorbents: injection of aluminum (Al) into sediments and thin-layer capping with Polonite (calcium–silicate), with and without the addition of activated carbon (AC), for their simultaneous sequestration of sediment phosphorus (P), hydrophobic organic contaminants (HOCs), and metals. Sediment cores were collected from a eutrophic and polluted brackish water bay in Sweden and incubated in the laboratory to measure sediment-to-water contaminant release and effects on biogeochemical processes. We used diffusive gradients in thin-film passive samplers for metals and semi-permeable membrane devices for the HOC polychlorinated biphenyls and polycyclic aromatic hydrocarbons. Al injection into anoxic sediments completely stopped the release of P and reduced the release of cadmium (Cd, −97%) and zinc (Zn, −95%) but increased the sediment fluxes of PAH (+49%), compared to the untreated sediment. Polonite mixed with AC reduced the release of P (−70%), Cd (−67%), and Zn (−89%) but increased methane (CH_4_) release. Adding AC to the Al or Polonite reduced the release of HOCs by 40% in both treatments. These results not only demonstrate the potential of innovative remediation techniques using composite sorbent amendments but also highlight the need to assess possible ecological side effects on, for example, sedimentary microbial processes.

## Abstract 2

Efficient sorbent amendments are urgently required for the remediation of contaminated soils and sediments. Here, we investigate new composite treatments that simultaneously decrease nutrients, hydrophobic organic contaminants , and metal fluxes.

## Introduction

Emissions of anthropogenic pollutants to natural waters in Europe have generally decreased due to stricter environmental regulations. Persistent legacy contaminants from historic industrial and agro-chemical sources are, however, still present at high concentrations in bottom sediments and can pose toxicity risks to bottom-living organisms and may constitute a contaminant source to the aquatic food web.^1^ An example of such internal contaminant loading is the redox-dependent phosphorus (P) release from Baltic Sea sediments that in spite of the reduced P input from land sources continues to drive eutrophication, with algal blooms and oxygen-depleted dead bottoms being the most acute symptoms.^2^ Ecotoxicological risks from hazardous substances such as hydrophobic organic contaminants (HOCs) and metals are high in the coastal areas of the Baltic Sea, and many such areas in Sweden will need remediation to achieve good status as demanded by the EU Water Framework Directive (WFD; 2000/60/EC).^3^

In situ sediment remediation is gaining attention as a potentially cost-efficient and less disruptive alternative to traditional ex situ treatment (i.e., dredging)^4^ and isolation capping.^5^ Thin-layer capping (TLC) in situ techniques involve sediment amendments with relatively small amounts of active sorbents—chemicals or materials that actively bind contaminants—that increase contaminant sequestration and consequently reduce mobility and bioavailability, to thereby promote a more rapid natural recovery of the sediment. Among the most utilized and studied sorbents for in situ remediation are lanthanum-modified clay (Phoslock)^6^ and various forms of aluminum (Al) addition^7,8^ for P sequestration, and activated carbon (AC) for the immobilization of HOCs.^9^ Many other sorbents have been evaluated in laboratory studies but are yet to be tested in large-scale field trials. These include variants of zeolite for the sorption of nutrients and metals^10,11^ and apatite and organoclays for metals and HOCs.^12,13^ Current literature reports no sorbent amendment, or mixture of materials, developed to simultaneously immobilize nutrients, HOCs, and metals.

In this study, we tested two recently proposed techniques for the in situ remediation of eutrophic, P-rich sediment: (1) injection of Al into sediments and (2) TLC with Polonite, an activated calcium silicate mineral. In addition, we tested the efficiency of these two treatments when amended with AC in order to create composite treatments that can sorb P, HOCs, and metals.

Al has been used to precipitate P in eutrophic lake water for decades.^7^ The sediment injection method is a new remediation technique thus far only applied in Sweden, where a solution of Al chloride is injected below the sediment surface, resulting in the formation of Al–P complexes.^8^ P is thereby sequestered in the sediment and unavailable for primary producers, which alleviates eutrophication by internal P loading. The method has been successfully used in lakes and a shallow coastal bay of the Baltic Sea.^14^

Natural minerals that are readily available at low cost are of interest as active components in TLC. One such example is zeolite that has been shown to sorb both nutrients and metals in laboratory studies.^11,15^ We investigated the sorbent Polonite for the same properties. Polonite is an activated (calcination at 900 °C) calcium silicate sorbent predominantly used in small-scale sewage treatment plants as a filter material (particle size 2–6 mm) with high capacity to bind P.^16^ It has been suggested to be useful as a TLC to treat P that has accumulated in Baltic Sea sediments.^17^ Moreover, Polonite is an alkaline material, which means it may also bind cationic metals through hydroxide precipitation and P co-precipitation, which can form insoluble precipitates.^18,19^ These properties make Polonite potentially suitable as a dual-purpose material for the remediation of sedimentary P and metal pollution. Our study tested a fine-grained fraction (0–0.5 mm) that is an abundant byproduct from the production of the commercial Polonite.

Many areas affected by eutrophication and metal contamination are also contaminated by HOCs. AC is a sorbent of HOCs that has been widely studied for use in sediment remediation.^9,20,21^ Large-scale field projects in Europe and the U.S. have shown that TLC with AC reduces the fluxes and bioavailability of HOCs such as polycyclic aromatic hydrocarbons (PAHs) and polychlorinated biphenyls (PCBs), which are present in the Baltic Sea and the Brunnsviken study area. When AC TLC has been applied in the field, its application has often included a second inert material such as a clean sediment, clay, or sand meant to function to further decrease HOC fluxes and AC material loss and increase the integrity of the active cap (i.e., to withstand erosion).^9^ The applicability and effectiveness of AC are already well studied. Hence, in the present study, we did not test AC only but aimed at investigating its performance with other sorbents. The composite amendments studied here were comprised only of active sorbents without mixing them with any inert material.

In order to test the sorbents under field-like conditions, we collected sediment cores from the polluted and eutrophicated Baltic Sea bay Brunnsviken in Stockholm, Sweden, and performed a 4 month experiment using sediment core incubations. The aims of our study were to investigate (i) the capacity of Al injection and Polonite to reduce the release of P from a nutrient-rich sediment, (ii) their ability to reduce the mobility of metals of environmental concern in the sediment–water interface, (iii) their performance in composite treatments with AC, for both P, metal, and sorption of HOCs, and (iv) potential physico-chemical and ecological side effects of these treatments, that is, on pH, exchange of greenhouse gases (methane), or effects on sediment microorganisms and the nutrient cycling processes they mediate.

## Materials and Methods

### Sediment Sampling

The study site Brunnsviken (Stockholm, Sweden, 59° 21′ 33.29″ N, 18° 2′ 59.91″ E, area 1.5 km^2^, maximum/mean depth 13.7:6.6 m) is a Baltic Sea bay in the inner Stockholm archipelago. It is an ideal site for studying eutrophication and pollution at the same time. Brunnsviken has a large urban catchment with residential, commercial, and industrial areas and is surrounded by heavily trafficked highways. Generally, contaminant accumulation in the sediments peaked around 1970 but is still ongoing (see Supporting Information, Section S4).^22^ The major driver of eutrophication is the historic deposition of phosphate (PO_4_^3^) in the sediment, where total P concentration in the surface sediment is between 1 and 1.5 g kg^–1^ dry sediment.^23^ Metals and HOCs such as PAHs and PCBs occur at concentrations exceeding sediment quality criteria set by the Swedish EPA.^22,24^ Inflows of freshwater from the catchment and brackish water from the archipelago create a halocline that reduces vertical mixing and oxygenation of bottom waters. In summer, a thermocline develops and the deepest areas of this enclosed bay generally become anoxic and sulfidic (euxinic) during the summer period (Supporting Information, Section S1).

Sediment was collected in March 2018 using a Kajak tube corer (⌀ 8, 50 cm length^25^) that was manually operated through holes drilled in the ice cover. In total, 20 sediment cores were collected from an accumulation bottom at 9 m depth. Surface water was collected in pre-cleaned polyethylene carboys and additional water was collected from Brunnsviken throughout the study to replace the overlying water in the tube cores (see below). In the lab at Stockholm University, the bottom stopper was carefully opened to remove some of the bottom sediment in order to get equal sediment and water heights in all cores (22 cm sediment and 26 cm water). The cores were then held in a dark climate chamber at 4 °C for 2 months, to acclimatize the sediments to atmospheric pressure and stop gas ebullition. Detailed descriptions of the sampling site Brunnsviken, initial sediment contaminant concentrations, the sampling, and experimental conditions are presented in Supporting Information, Sections S1 and S4.

### Sediment Treatments

The sediment cores (*n* = 20) were randomly allocated to five treatments: untreated control (CTRL), Al injection (AL), Al injection with AC TLC (AL + AC), Polonite TLC (POL), and Polonite and AC TLC (POL + AC).

#### Aluminum Sediment Injection

Previous lake remediation efforts in Sweden have injected sediments with polyaluminum chloride—an Al flocculent originally developed for wastewater treatment.^26^ It consists of an Al_2_Cl(OH)_5_ solution that is mixed with lake water before injection. The targeted sediment depth is 10–20 cm with a dose of 30–75 g Al^3+^ m^–2^.^8,14^ The PO_4_^3–^ content in the Brunnsviken sediment increases with lake depth.^23^ Al treatment dose should accordingly increase with depth, and following recommendations from previous studies a calculated dose of 80 g Al^3+^ m^–2^ was used here. This dose corresponded to 4.32 g of polyaluminum chloride (PAX XL-100, Kemira Kemi AB, Sweden) that was dissolved in 45 mL of Brunnsviken surface water and slowly injected at several places 8 cm into the sediment using a syringe with a 0.8 × 80 mm stainless-steel needle.

#### Thin-Layer Capping with Polonite

Renman et al.^17^ tested the P binding capabilities of Polonite in anaerobic Baltic Sea sediments. They used a composite cap of 75% powdered Polonite and 25% bentonite clay in 100 mL experimental units. An amount of 1 kg Polonite m^–2^ resulted in a 2–3 mm cap that reduced the flux of PO_4_^3–^ to the overlying water by 100% over a period of 12 weeks. Here, a dose of 1200 g m^–2^ powdered Polonite (0–0.5 mm grain size) was applied without bentonite to generate a 2–3 mm thick cap. 6 g of Polonite was suspended in 45 mL of Brunnsviken surface water and gently poured into the overlying water of the sediment cores. The material settled quickly and distributed evenly over the sediment.

#### Composite Amendments with AC

In addition to the treatments with either Al or Polonite, two new composite treatments were tested by adding a TLC of powdered AC (AquaSorb, BP2 PAC-S, *D*_50_ = 15–35 μm, Jacobi Carbons, Sweden) to reduce the sediment release of PCB and PAH. A relatively low dose of 600 g m^–2^ was selected to minimize adverse effects on sediment biota.^27^ A dose of 3 g of AC was mixed with 45 mL of Brunnsviken surface water into a homogenous suspension and gently poured into the overlying water of the core and left to settle.

### Sediment Core Incubations

After adding the treatments, the water column was replaced with new surface water from Brunnsviken to imitate the distinct annual mixing and oxygenation cycle in situ (Supporting Information, Section S1). When adding the water, a tightly fitted floating disc was placed inside the core to prevent sediment resuspension from water jets. Water changes before incubation 3 and 4 (see below) followed the same method. The room temperature was gradually increased from 4 to 16 °C, which is the approximate yearly temperature span in Brunnsviken bottom water (Supporting Information, Section S1), during a week before the first incubation was started. A magnetic stirring system (Supporting Information, Section S2) was installed following methods described by Quintana et al.,^28^ allowing a gentle continuous mixing (40 rpm) of the water column. In order to manipulate the oxygen level in the water column, each core was equipped with silicone tubing for air bubbling.

Five sediment core incubations were carried out under a total experiment duration of 135 days to assess the effects of treatments on sediment-to-water release of nutrients, contaminants, and gases under varying oxygen states (Table 1). For incubation measurements of nutrients and gases, we followed the protocol of Bonaglia et al.^30^ Briefly, water sampling was performed at the incubation start (*T*_0_). The cores were then sealed to create closed systems with continuous water mixing, and water sampling was repeated at the end of the incubations (*T*_F_). Water was collected from the center of the water column and filtered through a 0.45 μm filter. 15 mL was collected for analysis of phosphate (PO_4_^3–^), ammonium (NH_4_^+^), and the sum of nitrite and nitrate (NO_2_^–^ + NO_3_^–^ = NO_*x*_^–^) and refrigerated at 4 °C until chemical analyses. Fluxes were calculated from concentration differences of *T*_0_ and *T*_F_ (see Supporting Information, Section S3).

**Table 1:** Timeline and Experimental Conditions of the Five Incubation Experiments^c^

incubation no.	duration	days after treatment	oxygen state^b^	measurements
1^a^	8 h 40 min	7	oxic	PO_4_^3–^, NH_4_^+^, NO_*x*_^–^, O_2_, CH_4_
2	8 h 50 min	22	oxic	O_2_, CH_4_
3^a^	70 d	35–104	oxic, hypoxic	ΣPAH_16_, ΣPCB_7_, metals, PO_4_^3–^ using SPMD and DGT devices^b^
4^a^	3 d	125–127 (4 mo, 5 d)	oxic	O_2_
5	3 d	132–134 (4 mo, 12 d)	anoxic	PO_4_^3–^, NH_4_^+^, NO_*x*_^–^, CH_4_

a Replacement of the water column before incubation.

b Oxygen states were manipulated to mimic annual bottom water oxygen variations in Brunnsviken, that is, oxic, 7–10 mg O_2_ L^–1^; hypoxic, <2 mg O_2_ L^–1^; and anoxic, 0 mg O_2_ L^–1^.

c SPMD = semi-permeable membrane device passive sampling for HOC. DGT = diffusive gradients in thin-film passive sampling for PO_4_^3–^ and metals.

Oxygen (O_2_) concentrations were measured inside each sediment tube by microsensor reading (see Supporting Information, Section S3). pH was measured using a portable multimeter (HQ40d, Hach). Additionally, water was sampled for later dissolved methane (CH_4_) analysis and stored in 12 mL Exetainer glass gas-tight vials. Biological activity was stopped with a 200 μL dose of the zinc chloride solution (7 M), and samples were stored at 4 °C until analysis (1–2 weeks, see Supporting Information, Section S3).

#### 70 Day Incubation with Passive Samplers

A semi-permeable membrane device (SPMD) was installed in the water column to measure sediment-to-water fluxes of truly dissolved PAHs and PCBs. The SPMD is biomimetic, that is, it serves as an analogue to the lipid fraction of aquatic animals and can be used for assessing the bioaccumulation of HOCs in the environment and in laboratory experiments.^31^ We used 91.4 cm long, 2.5 cm wide SPMDs which were filled with ultra-high purity triolein (Environmental Sampling Technologies Inc., Missouri USA).

Diffusive gradients in thin-film (DGT) devices were used to measure sediment release of anionic PO_4_^3–^ and cationic trace metals. We used a DGT with two binding layers (LSNX-NP-loaded DGT, DGT-Research Ltd., England)^32^ to measure PO_4_^3–^, aluminum (Al), cadmium (Cd), copper (Cu), iron (Fe), manganese (Mn), nickel (Ni), lead (Pb), and zinc (Zn).

An incubation was initiated at day 35 with SPMDs and DGTs. The SPMD was mounted on a stainless-steel holder and hung with the DGT from a stainless-steel wire in the middle of the water column (Supporting Information, Section S2). All stainless steel was acid-bathed (0.1 M hydrochloric acid) to prevent the leaching of metals. The top rubber stoppers were replaced with stoppers made of cork (not air-tight) to allow some air exchange and to prevent possible contamination from the rubber stopper, which may leach phthalate and metals.^33^ The cores were incubated with magnetic stirring and no aeration for 70 days when hypoxia (<2 mg O_2_ L^–1^) had been achieved in all cores.

### Measurements of Sediment Vertical Profiles of O_2_, pH, and Sulfur

One core per treatment was sub-sampled using smaller core liners (⌀ 4.6 cm) and subjected to the high-resolution sediment profiling of O_2_, pH, and *S*_tot_ (H_2_S + HS^–^ + S^2–^), following the final incubation. Three profiles per parameter were recorded in each sub-sample. The method and calibration followed Marzocchi et al.^34^ Briefly, microsensors (O_2_: OX-50, *S*_tot_: SULF-50, pH: pH-100, Unisense, Denmark) attached to a digitally controlled micromanipulator recorded vertical profiles at sampling intervals of 100 μm for O_2_, and 250 μm for pH and *S*_tot_. The microsensors were connected to a four-channel multimeter (Unisense, Denmark).

### Chemical Analyses

Dissolved nutrients (PO_4_^3–^, NH_4_^+^, and NO_*x*_^–^) in sediment tube core water as well as sediment total P were quantified by means of colorimetric analysis; O_2_ with a pre-calibrated microelectrode (OX-500, Unisense, Denmark); CH_4_ using gas chromatography; sediment organic matter by means of loss-on-ignition; and sediment total organic carbon (C) and nitrogen (N) using an elemental analyzer. For details of these analytical methods, see the Supporting Information, Section S3.

The SPMDs were extracted and analyzed by means of gas chromatography–mass spectrometry for seven PCB congeners and their sum (ΣPCB_7_) and 16 PAHs (ΣPAH_16_). PAHs were classified according to the Swedish Environmental Protection Agency (NV) into three groups by molecular weight: light (PAH-L), medium (PAH-M), and heavy (PAH-H).^35^ Release fluxes of HOC (*F*_HOC_) within each core was calculated from the amount accumulated in the SPMD (*M*_SPMD_), the sediment surface area (*A*), and the deployment time (*t*), as proposed by Eek et al.^36^
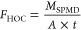
1

Extraction and analyses of accumulated HOCs in the SPMDs, as well as metals and PO_4_^3–^ accumulated in the DGTs, were done by the accredited laboratory at ALS Scandinavia (see Supporting Information, Section S3 for the description of analytical methods and quality assurance and Supporting Information, Section S4 for a complete list of analytes).

### Statistics

Differences between treatments were tested using ANOVA followed by Student–Newman–Keuls’ post-hoc tests. Levene’s test was used to assess the homogeneity of variances and Shapiro–Wilk’s test was used to assess normality. The robust Welch’s ANOVA was used in the case of heteroscedasticity. If normality was violated, a non-parametric Kruskal–Wallis test was performed, followed by Dunn’s pairwise comparisons (Bonferroni-adjusted *p*-values). All tests were performed in RStudio version 3.5.3 using packages car, ExpDes, dunnTest, and FSA. For statistical multiple comparisons between each treatment, see Supporting Information, Section S5.

## Results and Discussion

### Effects on pH

The control treatment (CTRL) experienced a drop in water column pH over the course of the experiment (Supporting Information, Section S6). The pH was 7.7 after the first incubation, and it had decreased to 6.9 by the end of the last incubation. This can be explained by H_2_S formation as oxygenation was reduced in later incubations.^37^

**Figure 1:**
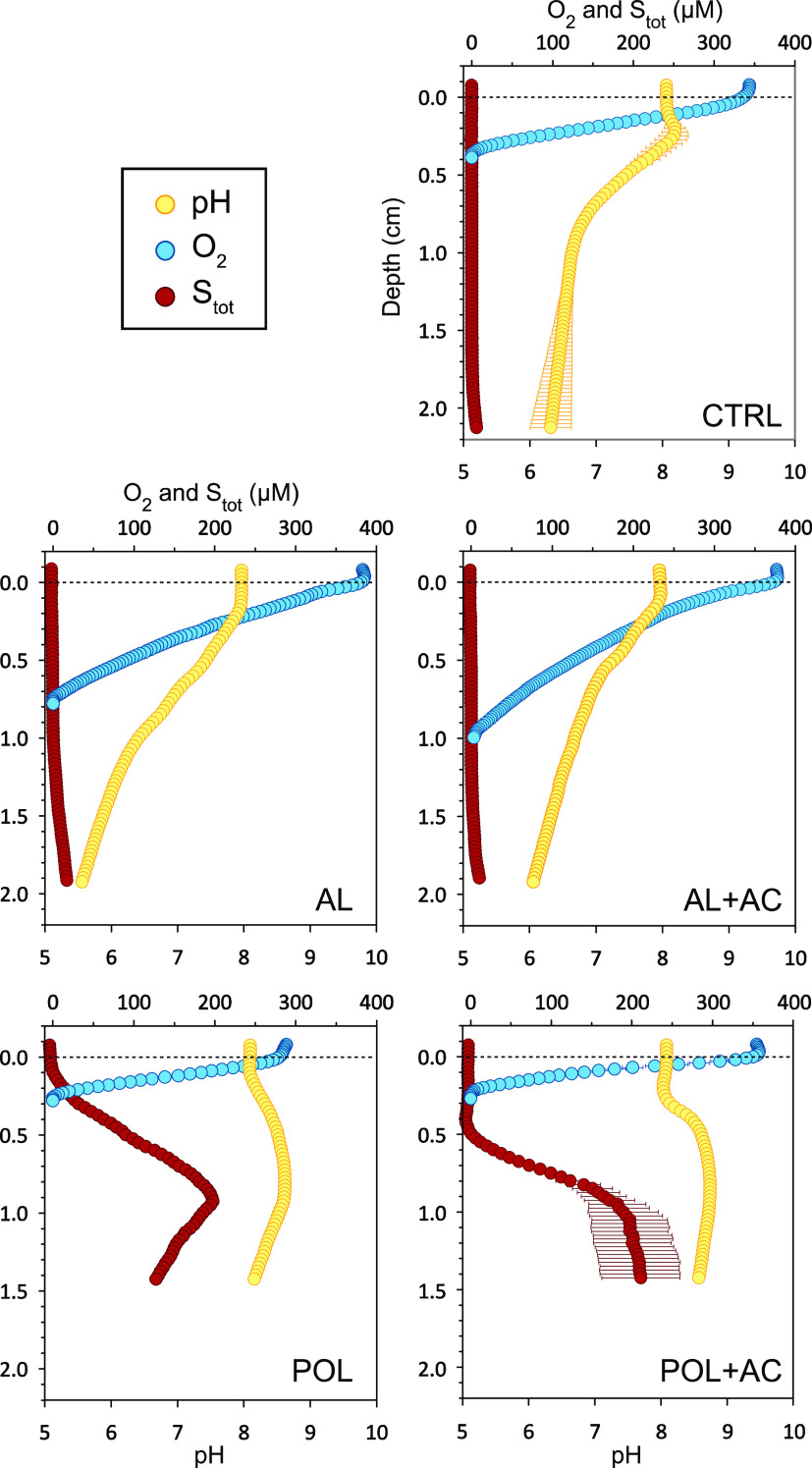
Sediment vertical profiles of pH, O_2_, and *S*_tot_ concentrations, after the final incubation. CTRL = untreated control; AL = Al injected into sediment; AC = sediment capped with AC, and POL = sediment capped with Polonite. Dots represent the mean of three profile measurements and error bars show standard error.

Water column pH in Al treatments was lowest at the end of the 70 day passive sampling incubation: pH 4.2 in AL and pH 5.3 in AL + AC, compared to pH 7.3 in CTRL. Sediment vertical profiling confirms that Al-treated sediments were more acidic than CTRL in the top 2 cm of the sediment (Figure 1). Capping with AC appears to have had a buffering effect on pH in AL + AC, which might be advantageous in the further development of this composite treatment. In earlier studies, when Al injection was applied in situ, the treatment was divided into several small doses over a long period of time. The treatment of the eutrophic Baltic Sea bay Björnöfjärden consisted of three injections spread over two summers, for a total dose of 50 g Al^3+^ m^–2^ injected at a 20 cm sediment depth.^14^ Their aim was to avoid acidification below pH 5.5 and consequential loss of PO_4_^3–^ binding capacity of polyaluminum chloride, mobilization of sediment metals, and toxic Al precipitation on fish gills.^24,38^ The effect of Al injection on pH was likely exacerbated in the present study that represents a worst-case scenario because the dose was applied as a single injection in a system with less pH buffering capacity than that of a lake. Al injection appears to have a long-lasting effect on pH: 9 years after treatment in lake Långsjön, water pH had decreased from an initial pre-amendment pH of 8.9 to 8.1. In lake Flaten, the pH had decreased from 8.5 to 8.3, 15 years after treatment.^8^ There is no previously published data on porewater pH, which based on this study should be monitored in future studies.

Capping with Polonite, on the other hand, increased both water and sediment pH because the material is alkaline.^19^ Water pH was 9.1 in POL and 9.0 in POL + AC 1 week after treatment (Supporting Information, Section S6). This pH increase diminished over the course of the experiment and toward the end of the experiment, Polonite treatments only exceeded control by 0.5 pH. However, sediment porewater pH remained elevated in the Polonite treatments by the end of the experiment, as shown by sediment vertical profiles (Figure 1).

### Effects on Phosphate Release and Nitrogen Cycling

**Figure 2:**
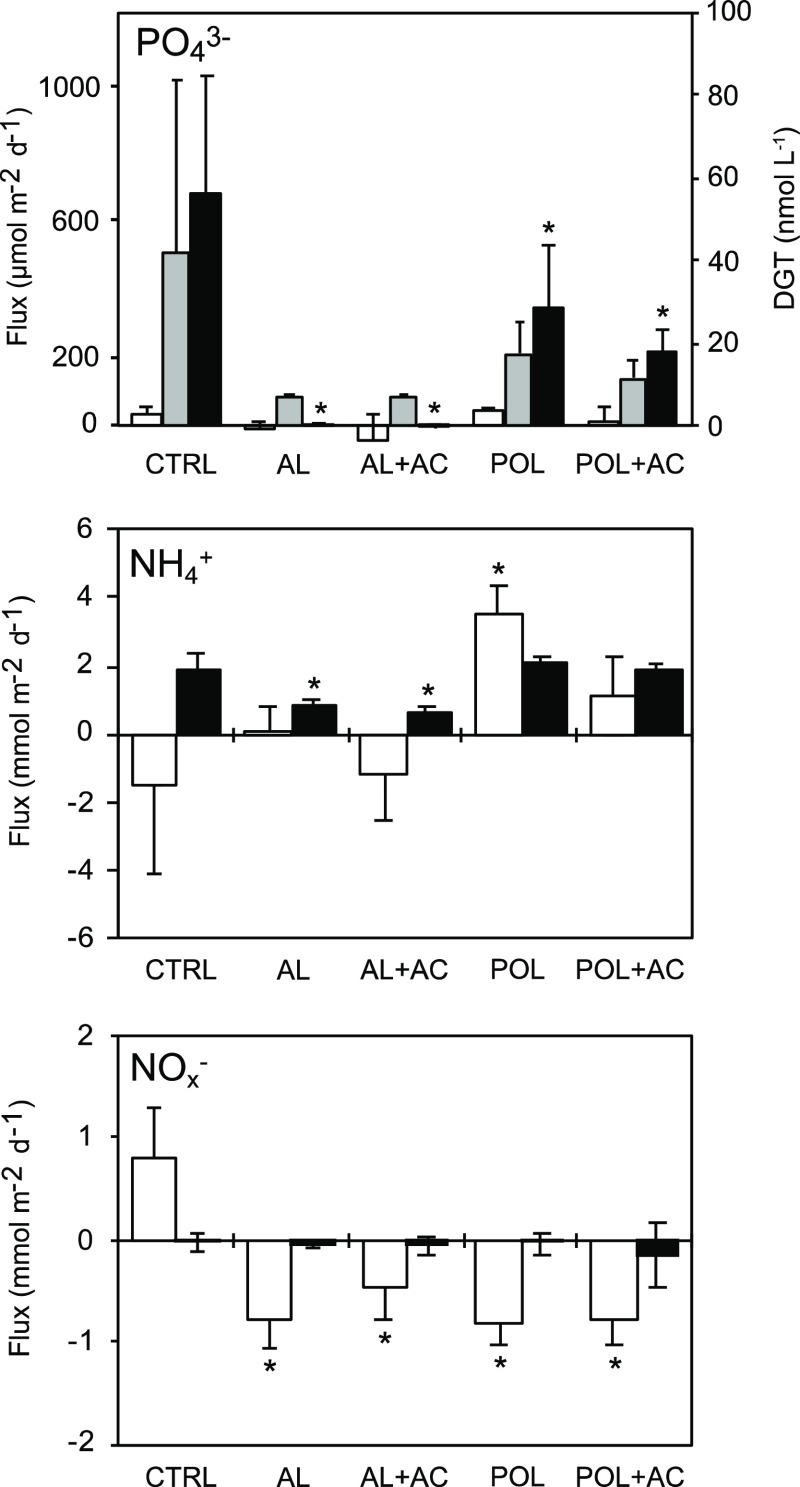
Fluxes of phosphate (PO_4_^3–^), ammonium (NH_4_^+^), and nitrite + nitrate (NO_*x*_^–^) measured in water samples 7 days after treatment under oxic conditions (white bars), and again 132 days after treatment in the anoxic incubation (black bars). PO_4_^3–^ was also measured by the DGT uptake (gray bars, right *y*-axis). CTRL = untreated control; AL = Al injected into sediment; AC = sediment capped with AC, POL = sediment capped with Polonite. Bars represent mean values ± standard deviation, *n* = 4. Asterisks denote significant difference from control (α = 0.05). For statistical multiple comparisons between each treatment, see the Supporting Information, Section S4.

Fluxes of PO_4_^3–^ were barely detected under oxic conditions and were highest under anoxic conditions in the untreated control (Figure 2). This corresponds well with the pattern observed in the Baltic Sea, where sediment deoxygenation results in phosphate release and contributes to the eutrophication of surface waters.^1,2^ Under anoxic conditions, every amendment significantly decreased the PO_4_^3–^ flux compared to untreated control sediments.

Under oxic conditions, AL and AL + AC showed slightly negative PO_4_^3–^ fluxes. This indicates that the injected Al was binding PO_4_^3–^ from the water column, but this effect could not be statistically verified. As the oxygen levels decreased during the longer 70-day incubation, the binding of PO_4_^3–^ to Al was effective but not complete because some PO_4_^3–^ accumulated in the DGTs. Both AL and AL + AC stopped the anoxic sediment-to-water flux of PO_4_^3–^ (0 μmol m–^2^ d–^1^), despite causing the acidification of the sediment and water, corroborating the results of previous studies on the efficacy of Al injection on P binding in sediments.^8,14^ The addition of AC did not impair the performance of the Al injection.

The 2–3 mm Polonite TLC reduced PO_4_^3–^ flux under anoxia by half (−49%). For comparison, a 2 cm zeolite TLC applied in a column experiment showed similar reduction in anoxic PO_4_^3–^ release (ca 60%).^11^ The composite TLC treatment of POL + AC showed even higher PO_4_^3–^ retention (−69%). AC has previously been shown to reduce PO_4_^3–^ fluxes in the Baltic Sea sediment, perhaps due to an Al content of 0.75% (here corresponding to 4.5 g Al m–^2^).^39^ The 1200 g m–^2^ Polonite dose produced a relatively thin^32^ 2–3 mm cap and would need to be increased to reach similar efficacy to the Al injection used in this study. Applying a thin-layer cap in situ is less complex than the repeated sediment injections, and Polonite TLC may represent a more cost-effective approach to mitigate eutrophication in systems with high internal PO_4_^3–^ loading or in lakes with low pH where Al injection is unsuitable.

The effects of Al and Polonite on PO_4_^3–^ fluxes measured with water samples under anoxic conditions are comparable to the effects measured with DGTs. However, the PO_4_^3–^ concentrations measured using DGTs showed a larger variance, which may be a consequence of natural variations in O_2_ saturation in the water column in the 70 day incubation period, compared to the shorter anoxic incubations which were maintained strictly anoxic by sealing the cores completely.

Fluxes of NH_4_^+^ were studied as a proxy for ammonification, that is, biological mineralization of organic nitrogen. Both AL and AL + AC showed significantly lower anoxic NH_4_^+^ fluxes than other treatments (Figure 2). In the absence of O_2_, this is interpreted as reduced ammonification, rather than increased nitrification.^40^ It is likely that microbial ammonification was reduced due to acidification in Al injection treatments.

The control sediment released NO_*x*_^–^ under oxic conditions, indicating that net nitrification occurred, whereas all other treatments had a negative flux of NO_*x*_^–^, which may suggest increased nitrate reduction processes (Figure 2). In sediment capped with Polonite, the NO_*x*_^–^ uptake was coupled with an increased NH_4_^+^ flux, which is indicative of dissimilatory nitrite/nitrate reduction to ammonium (DNRA).^40^ DNRA is an important part of N cycling, particularly in highly eutrophic sediments where NO_*x*_^–^ reduction is generally linked to the oxidation of hydrogen sulfide (H_2_S). It is likely that the increased concentrations of H_2_S observed from a 0.5 cm sediment depth of POL and POL + AC (Figure 1) further promoted DNRA. Increased NO_*x*_^–^ uptake in AL and AL + AC was not coupled to the increased NH_4_^+^ fluxes (and DNRA) and thus might have been transformed into N_2_(g) via denitrification or anammox. However, nitrification may have been inhibited by the low pH environment^41^ caused by Al addition. Because N cycling provides fundamental ecosystem functions that control primary production, these effects must be further studied.

### Effects on Oxygen Respiration and Methane Fluxes

**Figure 3:**
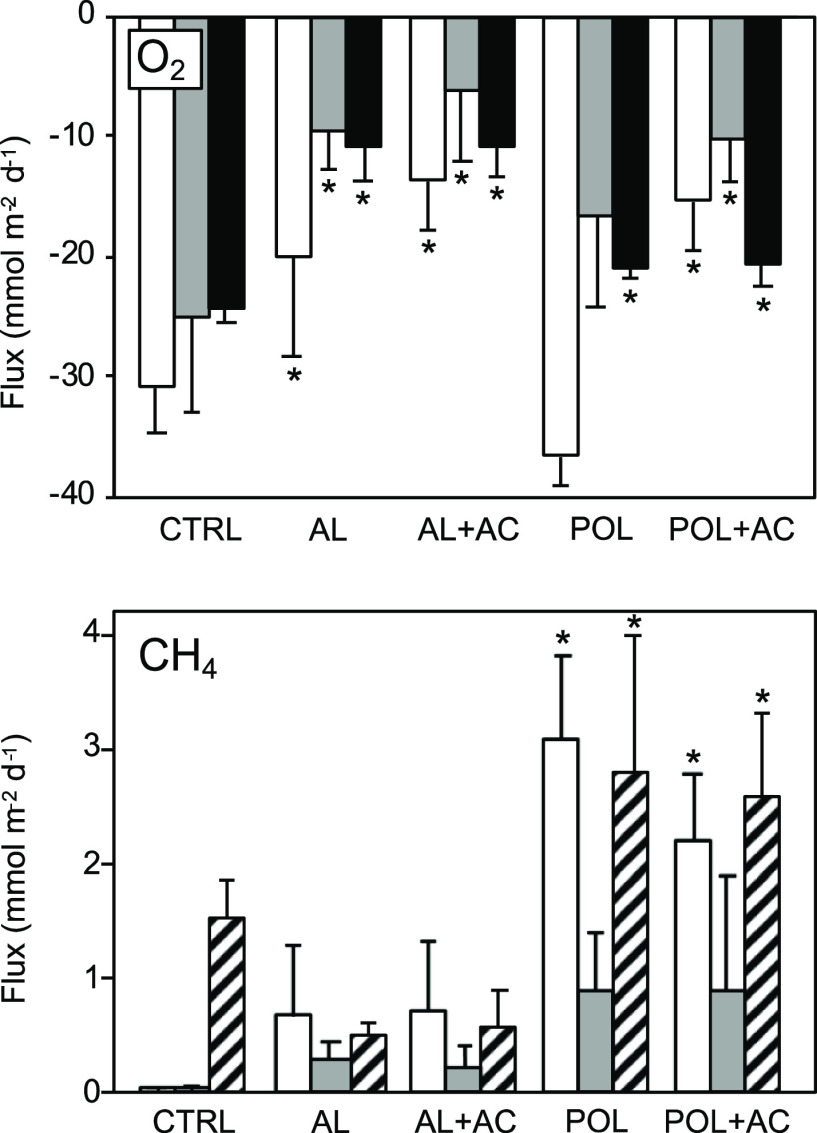
Fluxes of oxygen (O_2_) and methane (CH_4_) measured in sediment core incubations after 7 days (white bars), 22 days (gray bars), 125 days for O_2_ (black bars), and 132 days for CH_4_ under anoxia (striped bars). CTRL = untreated control; AL = Al injected into sediment; AC = sediment capped with AC; and POL = sediment capped with Polonite. Bars represent mean values ± standard deviation, *n* = 4. Asterisks denote significant difference from control (α = 0.05).

Oxygen flux (Figure 3) is considered a proxy for aerobic respiration by microorganisms and meiofauna, as no macrofaunal invertebrates were present in this sediment. Fluxes of O_2_ were negative in all incubations, that is, O_2_ was consumed. O_2_ consumption in AL and AL + AC treatments was significantly lower than in the control, indicating that AL had an inhibiting effect on aerobic respiration. Sediment O_2_ vertical profiles (Figure 1) showed that the oxygen penetration depth was greater in AL (0.76 cm ± 0.04 cm) and AL + AC (1.04 ± 0.06) than in the control (0.38 ± 0.02 cm), indicating that O_2_ consumption was lower in sediments treated with Al injection. This is in line with a reduced microbial ammonification in Al treatments. These results suggest that Al injection caused acute adverse effects on the microbial community, likely due to the acidity of the material, as pH is known to affect the abundance and species diversity of microbial communities in sediments.^42−44^

Decreased respiration was consistently observed in POL + AC, while in POL the decrease was only observed in the last POL incubation, suggesting that AC may also have adverse effects on oxygen-consuming microbes or act as an oxygen sink, the latter having been observed previously.^45^

In oxygen-depleted sediments, certain microbes can utilize CO_2_ and other carbons as electron acceptors for their respiration.^46,47^ These specialized microbes, called methanogens, produce CH_4_. Accordingly, CH_4_ fluxes in our control treatment only occurred during anoxia (Figure 3). Surprisingly, every amendment showed that CH_4_ release was not limited to anoxic conditions. Treatments AL and AL + AC had higher fluxes than the control under oxic conditions but not significantly higher. Substantial fluxes also occurred during the first oxic incubation in POL and POL + AC. Hypothetically, capping with Polonite may have triggered the vertical diffusion of CH_4_ through sediment compaction. It is also possible that the alkalinization observed in Polonite treatments caused higher CH_4_ fluxes. As mentioned earlier, pH is a fundamental controller of the sediment bacterial community structure and abundance. The increased sediment pH following Polonite addition might have caused a shift in the microbial community toward alkaliphilic methanogens.^48,49^ When looking at the sediment vertical profiles for *S*_tot_ (H_2_S + HS^–^ + S^2–^, Figure 1), skewing was seen in treatments POL and POL + AC, where *S*_tot_ concentrations were increased below the oxygen penetration depth. The occurrence of *S*_tot_ is an indicator of microbial sulfate (SO_4_^2–^) reduction which may occur together with methanogenesis in sediments with high organic load.^50,51^ Moreover, in the presence of acetate or lactate, which are available in nutrient-rich systems such as Brunnsviken,^52,53^ the rate of both methanogenesis and sulfate reduction is increased.^54^ Phelps and Zeikus^55^ performed a series of experiments on the anoxic lake sediment and found a positive relationship between increased pH (from pH 6 to 7) and methanogenesis via CO_2_ reduction. Interestingly, they also showed that a *S*_tot_ concentration of 220 μM was optimal for methane production. In our results, the highest *S*_tot_ concentrations in POL and POL + AC were ca 200 and 210 μM, respectively (Figure 1).

We hypothesize that pH changes caused a shift within the microbial community structure toward higher abundances of bacterial consortia performing sulfate reduction that in turn or in part increased methanogenesis. More studies are needed to investigate how Polonite and other calcareous materials proposed for TLC relate to methane release and *S*_tot_ formation, and how this may affect other essential biogeochemical processes.

### Effects on the Release of PCBs and PAHs

**Figure 4:**
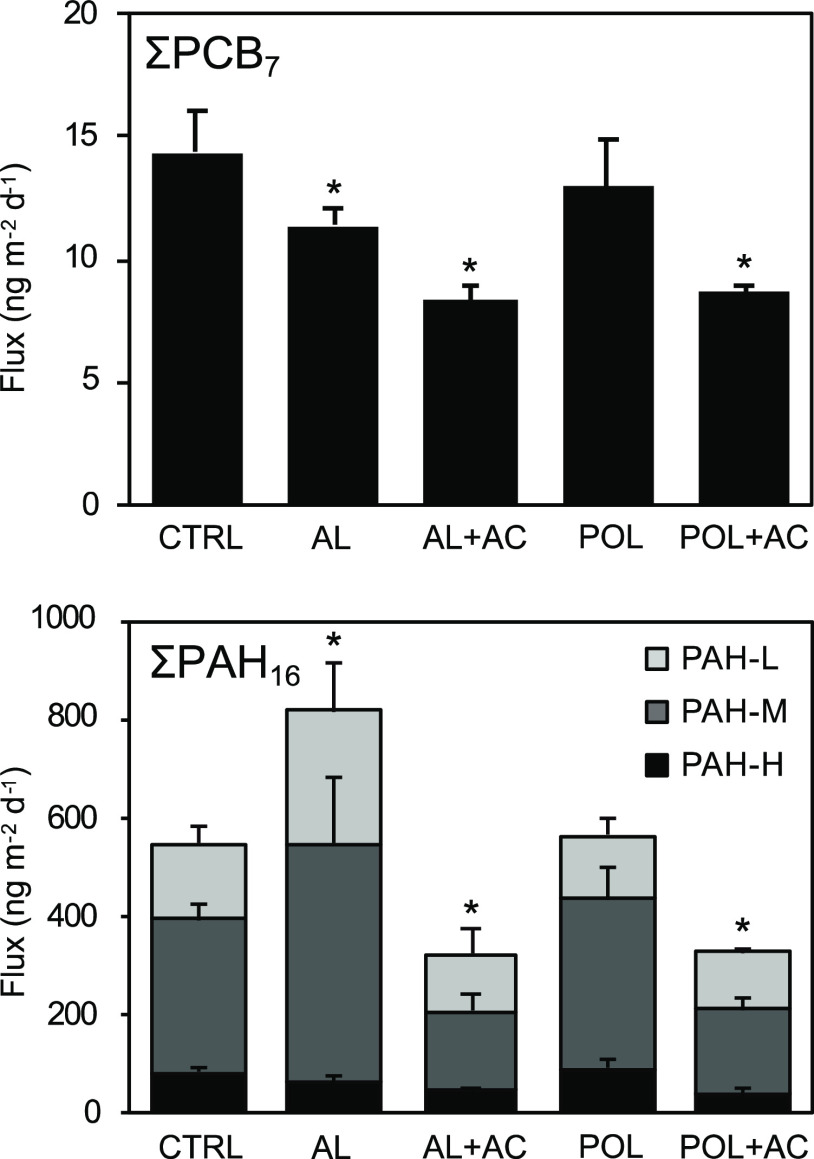
Fluxes of ΣPCB_7_ and ΣPAH_16_ comprising three molecular weight classes: light (PAH-L, light gray bars), medium (PAH-M, dark gray bars), and heavy (PAH-H, black bars) congeners. CTRL = untreated control; AL = Al injected into sediment; AC = sediment capped with AC, and POL = sediment capped with Polonite. Bars represent mean values ± standard deviation, *n* = 4. Asterisks denote significant difference from control (α = 0.05).

The sediment release flux of ΣPCB_7_ decreased in AL (−21%), AL + AC (−42%), and POL + AC (−40%) treatments in relation to the control (Figure 4). Polyaluminum chloride has previously been shown to sequester PCB when used as a coagulant to treat drinking water,^56^ while our data are the first to show the effects of Al injection on the attenuation of PCB release from the sediment. Capping with Polonite only (POL) did not have an effect on HOC release. AC amendment reduced ΣPAH_16_ fluxes (−41% for AL + AC and −40% for POL + AC) but it was not effective on PAH-L, the lightest weight class. This may be explained by the positive relationship between the PAH molecular weight and organic carbon–water partition coefficient, log *K*_OC_, that is, the lighter PAH molecules have a lower affinity for organic carbon (Supporting Information, Section S4) and therefore AC.^57^

Surprisingly, Al injection without AC led to a 49% increase in the release flux of ΣPAH_16_ (Figure 4). When examining PAH weight classes by pairwise comparisons (Supporting Information, Section S5), the increased flux is attributed to PAH-L (+74%) and PAH-M (+56%), whereas PAH-H was unaffected, likely due to their higher log *K*_OC_. It is unclear whether decreased pH is the primary driver behind the increased PAH release, as both positive^58,59^ and negative^60−64^ relationships between pH and PAH sorption to sediment organic matter have been reported. Secondary effects from acidification are also plausible. For instance, microbial mineralization and degradation of PAHs might have been slowed by the low pH, as has been shown for naphthalene (a PAH-L),^65^ thereby making larger quantities available for release.

It is also evident that the ionic strength (ion concentration) was increased by the AL treatment, due to increased concentrations of Al^3+^ and dissolution of other metal ions (caused by low pH, discussed below). Ionic strength is a key controlling factor for sediment HOC mobility, but the mechanisms and relationships are intricate and not yet fully understood.^61,66,67^ Finally, free Al^3+^ ions may have outcompeted PAH-L and PAH-M in binding to humic substances. This idea is supported by studies that describe the uniquely high affinity of Al^3+^ to humic substances.^68−70^

### Effects on Metal Release

**Figure 5:**
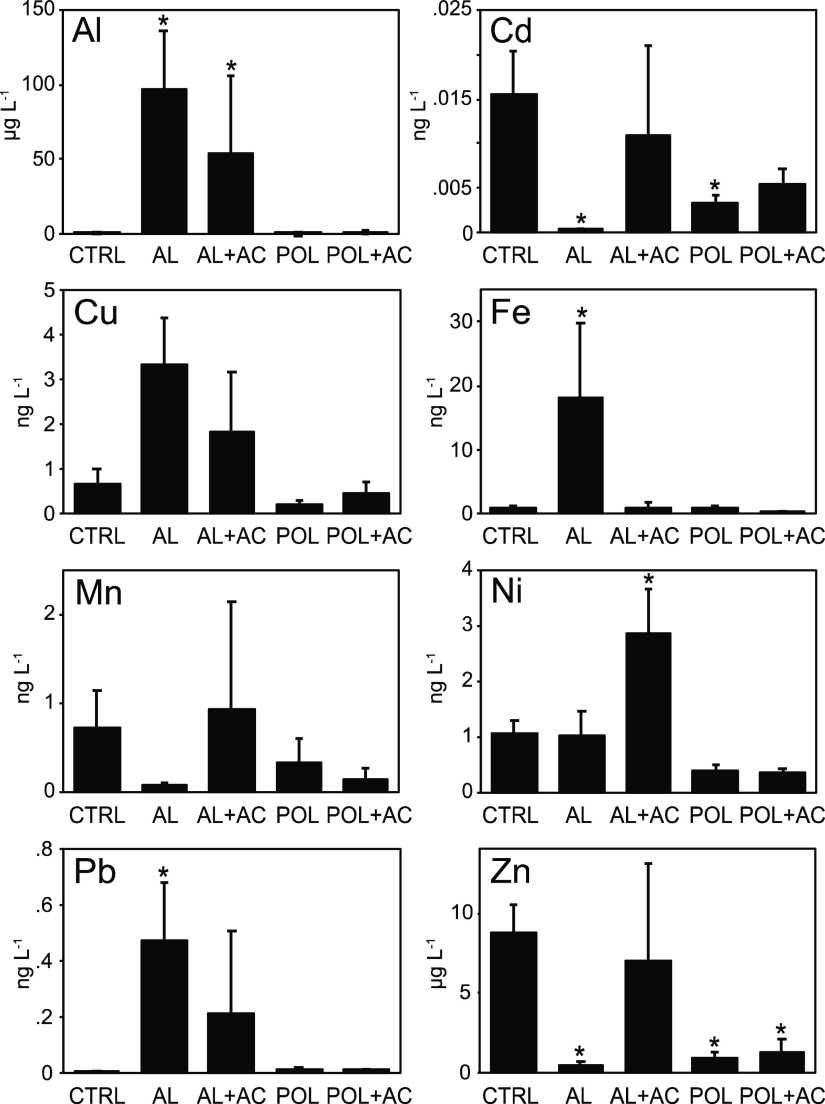
Average water concentrations of metals, as estimated by the DGT uptake. CTRL = untreated control; AL = Al injected into sediment; AC = sediment capped with AC; and POL = sediment capped with Polonite. Bars represent mean values ± standard deviation, *n* = 4. Asterisks denote significant difference from control (α = 0.05).

Metal concentrations in water under oxic–hypoxic conditions are shown in Figure 5. Both treatments with Al injection increased Al concentration, and the AL treatment strongly reduced Cd (−97%) and Zn (−95%) but increased Fe (1700%) and Pb (5600%) water concentrations. The AL + AC treatment did not increase Fe but increased the concentration of Ni (270%) compared to the control and AL treatments. The increased Fe and Pb concentrations in the AL treatment are not surprising as pH decreased in both water (Supporting Information, Section S6) and sediment (Figure 1) in that treatment. Cationic metals, studied here, associate with and may be immobilized by a number of different sediment constituents, as reviewed by Bryan and Langston.^71^ Hence, there are many factors controlling the speciation and mobility of metals, and pH is essential.^72^ Generally, metals dissociate in their bonds when pH decreases and may diffuse across the sediment–water interface or bioaccumulate.^73,74^ While the release of Al and Fe increased in the low pH of the AL treatment, Cd and Zn were immobilized, suggesting that other effects may govern their speciation. Previous in situ Al injection treatments have shown no indications of harmful metal release from the sediment (further discussed in Supporting Information, Section S7).

The treatment with Polonite reduced water concentrations of Cd (−67%) and Zn (−89%), that is, the 2–3 mm Polonite TLC was slightly more efficient than the 2 cm zeolite TLC described by Xiong and co-workers.^11^ The POL + AC treatment had no additional effect on metal sequestration.

### Environmental Implications and Future Perspectives

The sorbents and new composite treatments successfully immobilized the respective target contaminants. Both Al injection and Polonite TLC applied together with AC led to the effective sediment sequestration of PO_4_^3–^, HOCs, and metals—a uniquely broad range of contaminants in the context of TLC. The results indicate that the already established Al injection method can be modified by adding AC to obtain a composite treatment for a eutrophic and contaminated sediment. A successful remediation against eutrophication and subsequent oxygenation of the bottom would facilitate the re-establishment of benthic fauna, which in turn can increase the release of contaminants via bioturbation^33,75^ or promote bioaccumulation and biomagnification. Hence, Al injection with AC can be suitable for use on euxinic bottoms with extremely poor fauna communities, such as in Brunnsviken,^76^ especially because high doses of powdered AC can have adverse effects on biota.^27,77^ The application methods of Al injection and AC TLC in situ are well developed and could be combined to produce an amendment with long-lasting positive effects. In lake Flaten, Al injection was done 20 years ago, and the ecological status of the lake is now high.^78^ Amendment with AC is more recent so no long-term data are available yet. However, remediation in Grenlandsfjorden was effective in the sequestration of dioxins and furans (PCDD/Fs) for up to a decade after treatment.^79^

The mechanism behind the increased release fluxes of light and medium weight PAHs observed in the treatment with Al only has not been reported before. Further studies are necessary to measure potential harmful release of PAHs in contaminated systems remediated with Al. Sorption experiments with varying pH should be conducted to distinguish the roles of pH and Al^3+^ in the mobilization of HOCs. We advocate the monitoring of PAHs, before, during, and after Al injection in remediation projects, for example, by deploying passive samplers in the field.

Our results suggest that Polonite can be used in TLC as a sorbent for P and metals. Because alkalinity is the basic property of Polonite that enables metal sorption, future studies, ideally in the field, should assess any long-term pH-dependent side effects on the benthic ecosystem and investigate the increased methanogenesis observed here. If the Polonite would prove inadequate for use on anoxic bottoms because of this, its potential use in oxic environments should be considered. In situ application of the Polonite TLC would resemble that of AC, for example, slurry application^9^ and be cost-efficient because the material is an abundant byproduct.

This study highlights the potential of innovative composite sorbent amendments for remediation aimed at several target contaminant groups. There is no remediation method suitable for all polluted sediments, but composite treatments can provide high site-specificity and more targeted remediation. The composite treatments proposed here may replace or supplement expensive and ecologically disruptive remediation techniques, such as dredging and isolation capping.

## Supporting Information Available

Map of Brunnsviken and surroundings, monitoring data, sediment core sampling and photographed, three random cores sampled, concentrations of nutrients in surface water, experimental setup, sediment surfaces, illustration showing one sediment core of each treatment, organic contaminant PAHs, sediment vertical profiles, overview of measured parameters, evolution of pH with time, and time series (PDF)
