# Supplementary material for: Sediment Remediation with New Composite Sorbent Amendments to Sequester Phosphorus, Organic Contaminants, and Metals
Source: Environ Sci Technol. 2021 Aug 26;55(17):11937–47. doi: 10.1021/acs.est.1c02308 (PMC8427744; doi:10.1021/acs.est.1c02308)
Supplement: Supplementary file 1 — es1c02308_si_001.pdf [file es1c02308_si_001.pdf]

Supporting Information for:

Sediment remediation with new composite sorbent amendments to sequester phosphorus, organic contaminants and metals.

Johan Wikström[1*], Stefano Bonaglia[1,†], Robert Rämö[1], Gunno Renman[2], Jakob Walve[1], Johanna Hedberg[1], Jonas S. Gunnarsson[1]

[1]Stockholm University, Department of Ecology, Environment and Plant Sciences (DEEP), 106 91 Stockholm, Sweden

[2]KTH Royal Institute of Technology, Department of Sustainable Development, Environmental Sciences and Technology, Division of Water and Environmental Engineering, 100 44 Stockholm, Sweden

[†]Present Addresses: University of Gothenburg, Department of Marine Sciences, 405 30 Gothenburg, Sweden.

[*]Corresponding author, e-mail address: johan.wikstrom@su.se

Number of pages: 14
Number of tables: 5
Number of figures: 11

Table of contents:

Section S1 – Brunnsviken Sampling and Water Chemistry pg. 2-4
Section S2 – Experimental Setup pg. 4-6
Section S3 – Incubations and Chemical Analyses pg. 6-9
Section S4 – Sediment Characteristics and Contaminant Conc. pg. 10-12
Section S5 – Details of ANOVA and Pairwise Comparisons pg. 12
Section S6 – Water Column pH pg. 13
Section S7 – Effects of Aluminum Injection on Metal Release pg. 13
References pg. 14

SECTION S1 – BRUNNSVIKEN SAMPLING AND WATER CHEMISTRY

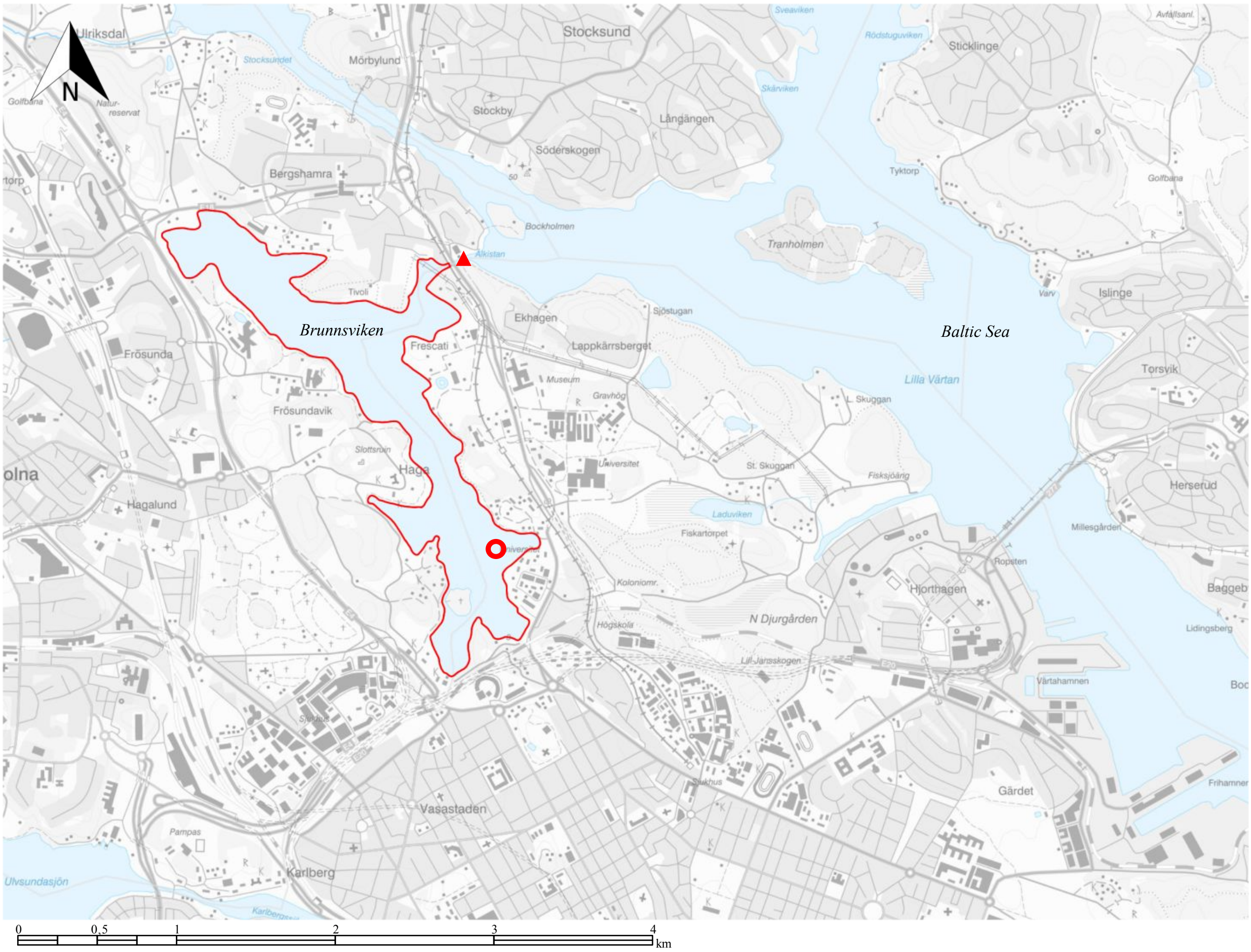

Figure 1. Map of Brunnsviken and surroundings. Red circle shows the sampling location (59° 21' 33.29" N, 18° 2' 59.91" E). Notice the narrow outlet to the Baltic Sea in the northeast corner of the bay, marked by the triangle. The map was modified from an original from the Swedish Cadastral Registry (Lantmäteriet).

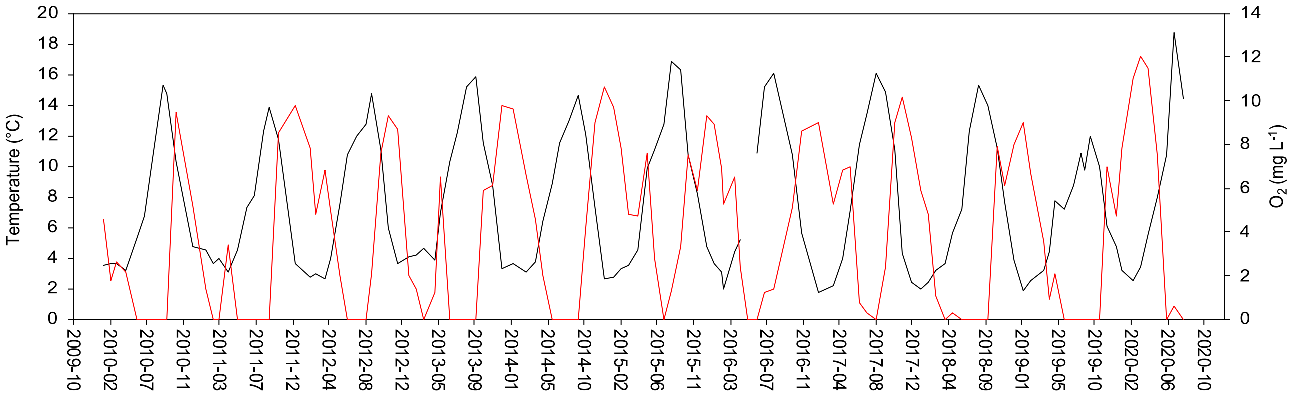

Figure 2. Monitoring data between 2010 and 2020 over temperature (black line) and oxygen concentration (red line) at 8 m depth. Data was collected ca 150 m northwest from our sampling site by SVOA.[1]

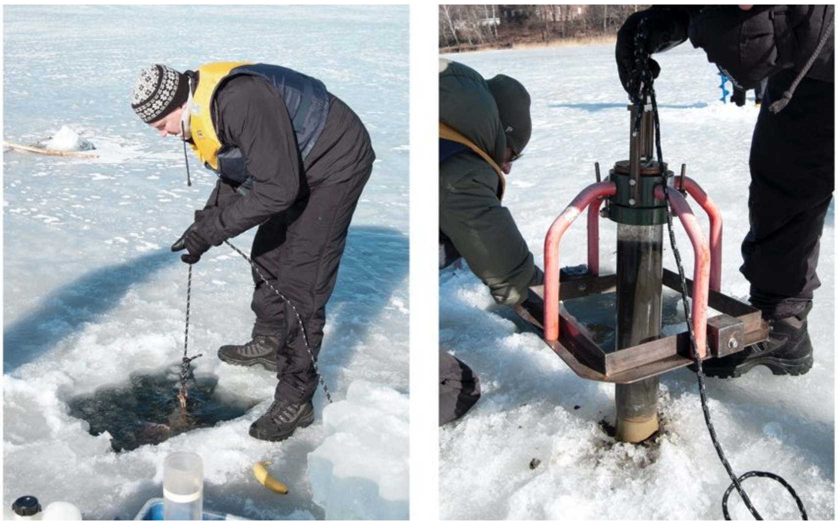

Figure 3. Sediment core sampling through the 30 cm thick ice of Brunnsviken. The Kajak tube corer was lowered manually to 9 m depth.

Sampling took place in March of 2018 from the ice of Brunnsviken, since sampling of the very loose sediment from boat during summer has been proven a challenge.[2] Sampling through the ice with a Kajak corer modified for soft bottoms[3] was an effective approach. The retrieved sediment cores had intact sediment-water interfaces and a clear, anoxic, water column on top owing to the absence of wind-driven circulation and low particle resuspension below the ice during winter. The collected sediment cores were transported to Stockholm University and stored in darkness at 4°C. A two-month period of acclimatization to the new conditions was initiated to let some of the gas within the sediment escape and to re-oxygenate the water. Three of the cores were randomly selected for monitoring of water chemistry during this period (Table 1) before any replacements of the overlying water had been made. The water had very high concentrations of nutrients, and also large variability between the cores. Most likely, fluxes of nutrients from sediment to overlying water was facilitated by resuspension due to gas ebullition and handling of the cores. The temperature was slowly increased to 16°C before experimentation began, to imitate in situ temperature fluctuations (Figure 2).

Table 1. Three random cores were sampled for core water nutrient concentrations during the two months long acclimatization to atmospheric pressure.

**Random core**	**Date of sampling**	**Days until experiment start**	**PO_4^{3-} µmol L^{-1}**	**NH_4^+ µmol L^{-1}**	**NO_X^- µmol L^{-1}**
1	2018-04-10	36	13,6	129	10,7
	2018-04-11	35	13,5	129	9,7
	2018-04-12	34	13,6	136	8,9
	2018-04-13	33	13,9	144	8,0
2	2018-04-10	36	23,4	270	0,6
	2018-04-11	35	24,2	280	0,6
	2018-04-12	34	24,5	288	1,2
	2018-04-13	33	25,8	302	0,5

3	2018-04-10	36	23,5	236	0,5
	2018-04-11	35	21,8	245	0,5
	2018-04-12	34	19,6	244	0,5
	2018-04-13	33	19,4	261	0,5

The water that was collected from Brunnsviken and used to replace the core water on three occasions was also analyzed for nutrient concentrations (Table 2). Dissolved nutrients were generally lower than in the original core water. The water replacements were done to imitate mixing and oxygenation of the water column that occur in spring and fall in situ (Figure 2), and to remove any pre-equilibrated analytes before the incubations. In this manner, we aimed to maintain in situ conditions in the lab, in order not to alter the natural biogeochemical processes we wanted to study.

Table 2. Concentrations of nutrients in surface water that was collected from Brunnsviken on three occasions and used to replace the water in the cores.

Date of collection	Experiment day	PO_4^{3-} µmol L^{-1}	NH_4^+ µmol L^{-1}	NO_X^- µmol L^{-1}	Water replacement
2018-05-21	5	0,07	2,59	0,84	Before incubation 1
2018-05-21	5	0,11	3,19	0,83	Before incubation 1
2018-06-13	28	0,10	3,16	0,18	Before incubation 3
2018-09-04	111	1,01	4,74	2,06	Before incubation 4

The alkalinity of Brunnsviken is high, at 85 ± 6 mg HCO_3 L^{-1} (mean of two sites, ± 1 standard deviation, in surface water measured monthly from August 2019 and one year ahead),[1] and buffering capacity against pH changes is considered good.

SECTION S2 – EXPERIMENTAL SETUP

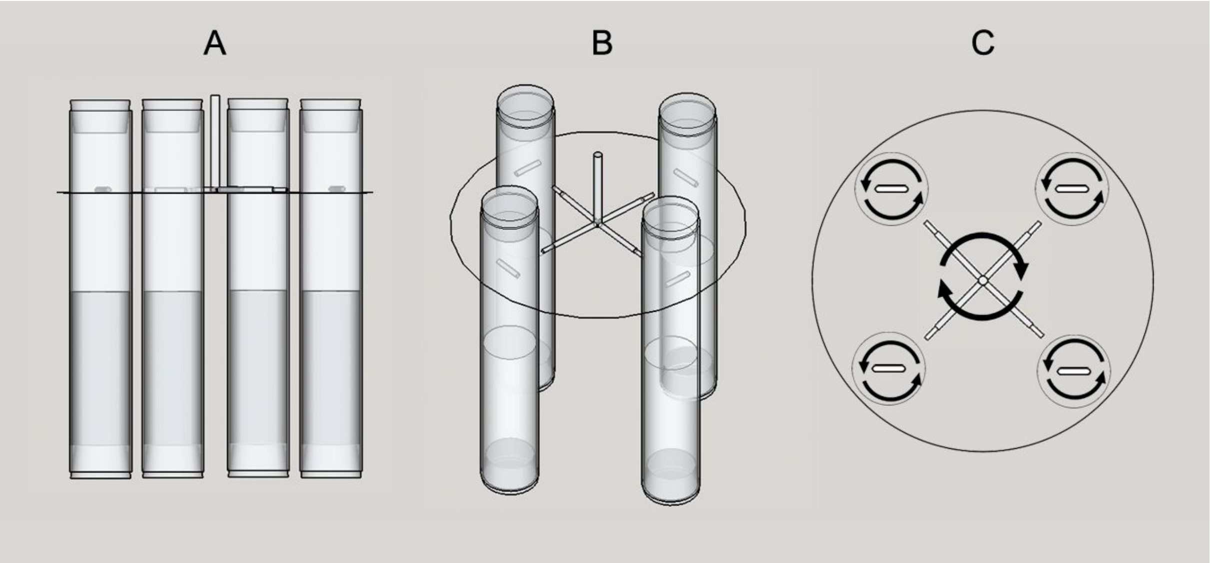

Figure 4. Schematic presentation of the experimental setup. **A**: Sideview of a stand holding sediment cores with rubber stoppers at top and bottom. The actual setup had 6 – 7 cores per stand. **B**: Overview of a stand with four sediment cores, one magnet bar in each core and the magnetic stirrer arms driven by a top mounted motor (not shown). **C**: Top view of the magnetic stirring system with central rotating arms complete with magnets at the ends that drive the magnetic stirrers installed in each core.

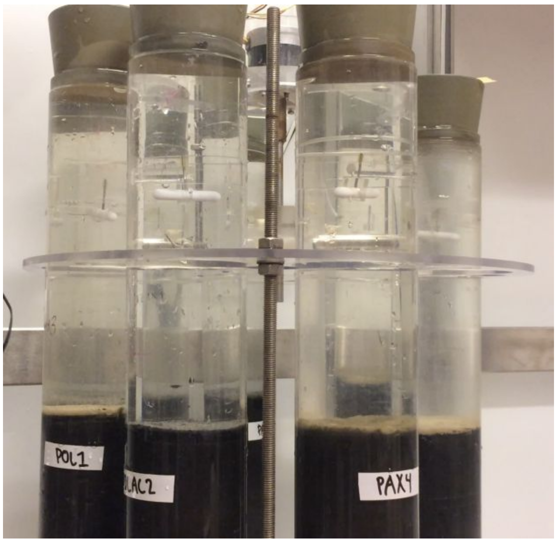

Figure 5. Sediment cores photographed before the first incubation. Rotating magnets are seen in the water column and treatment sorbents visible on top of the sediment.

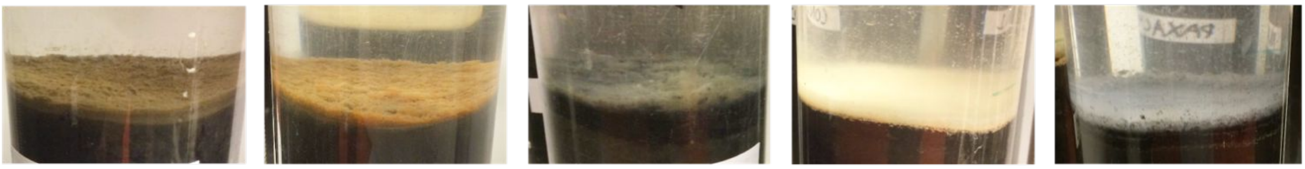

Figure 6. Sediment surfaces in the cores five weeks after adding the sorbents. From left to right: untreated control (oxidized top layer); Al injection (brownish floc had emerged from sediment); Al injection + AC TLC; Polonite TLC; Polonite and AC TLC.

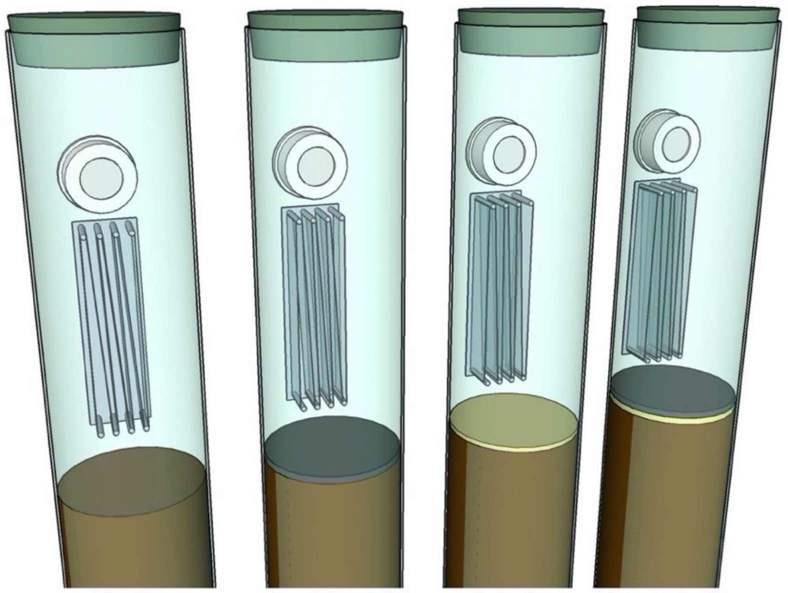

Figure 7. Illustration showing one sediment core of each treatment (control not included) and the passive samplers within the cores. The white cylinders depict the DGT-samplers, and below are the transparent SPMDs, wound around metal holders. (Not in scale).

SECTION S3 – INCUBATIONS AND CHEMICAL ANALYSES

Sediment core incubations were done to measure fluxes of nutrients and gases. Flux is defined as the flow rate per unit area across the sediment-water interface:

$$Flux = \frac{(C_{T_F} - C_{T_0}) \times V}{A \times t} \times 10\,000 \qquad (1)$$

Where C_{T0} and C_{TF} are the water concentrations at the start and end of the incubation, respectively, t is the incubation duration, A is the sediment surface area, and V is the water volume. The sediment surface area was 50.24 cm^2, the water column height was 26 ± 0.65 cm, and the core water volume was 1.3 ± 0.03 L.

O_2 measurements in the water column were done with an oxygen microelectrode (OX-500, Unisense A/S, DK) for quick readings (90% response time < 3 s). Calibration slopes were acquired by measurements of 100% saturated (5 min strong air bubbling) and 0% saturated (5 min strong N_2 bubbling) Brunnsviken surface water. In water with salinity of 2 and at 16 °C, O_2 has a solubility of 302.7 µmol L^{-1}.[4] The readings were then converted to oxygen concentration. Water pH and salinity were monitored using a multimeter (HQ40D, Hach).

The analysis of dissolved CH_4 concentrations was performed by headspace-gas chromatography, according to Sithersingh & Snow,[5] by injection of 1 mL headspace into a Shimadzu 8A gas chromatograph (GC) equipped with a flame ionization detector (FID). The calibration was performed by repetitive injections of 1 mL certified standard gases (11.2 ppm CH_4, Air Liquide, France). A

solubility coefficient for CH_4 in water with salinity 2 was used to calculate the water concentration of CH_4.[6]

Dissolved nutrients in the water column (PO_4^{3-}, NH_4^+, NO_X^-) were analyzed at the accredited chemical lab at DEEP, Stockholm University, by colorimetric analysis on a segmented flow nutrient analyzer system (OI Analytical, Flow Solution IV, Xylem Inc., USA, method #319528 for PO_4^{3-}, method #319526 for NH_4^+, and method #319527 for NO_X^-).

Extraction and analyses of passive samplers

For HOC and metal data, we used upper-bound values, i.e., values that were reported as <LOD (limit of detection) were included as the LOD value, and not set to zero (lower-bound). This was done in order to not overestimate the efficiency of the treatments, or underestimate the contaminant release.

SPMD extraction and analysis

Performed by ALS Scandinavia following the conditions and requirements of the US EPA method 429 (ISO11338) for PAHs, and method 1668 for PCBs, briefly described here. The SPMDs were cut open and the content was spiked with stable isotopically labelled PAH/PCB congeners and extracted three times with n-hexane. After cleaning the extracts through multilayer silica columns, they were concentrated using a Kuderna-Danish concentrator and finally spiked with injection standard before quantification using GC/HRMS:

PAH measurement conditions:

Finnigan MAT 95XP/ Agilent Technologies GC 6890N.
HRMS: Resolution: ≥ 8000; EI ionization
Column type: Rxi - 17MS (30 m x 0.25 mm, film 0.25 µm)

PCB measurement conditions:

Thermo DFS/ TRACE GC Ultra Series
HRMS: Resolution: ≥ 10 000; EI ionization
Column type: MXT-500 (60 m x 0.25 mm, film 0.15 µm)

DGT extraction and analysis

Performed by ALS Scandinavia following these steps: the samplers were disassembled, and the binding layers were leached in 10 ml 10 % nitric acid (HNO_3) over night. The solutions were then diluted five times to 2 % HNO_3 before analysis by inductive coupled plasma sector field mass

spectrometry (ICP-SFMS). The DGT equation[7] was used to calculate time-weighted average water concentration of the analytes.

Metal measurement conditions:

The measurements were carried out using an Element (Thermo Scientific, Bremen, Germany) sector field ICP-MS (ICP- SFMS) instrument equipped with introduction system comprising PFA MicroMist nebulizer, a Peltier-cooled PFA spray chamber (Elemental Scientific Inc., Omaha, NE, USA), demountable torch with a 1.5 mm quartz injector, nickel sampler (1 mm orifice) and skimmer (0.8 mm orifice) cones. See references 5 and 6 for details on operation conditions and measuring parameters.

Brunnsviken sediment characteristics

At the end of the experiment, three of the control cores were sub-sampled and sectioned at a sediment depth of 2-4 cm and used for the chemical analyses described below (contaminant concentrations, total organic carbon, total phosphorous, total nitrogen, water content and loss on ignition). The sediment samples were kept frozen (-20 ^{0}C) until analysis.

Sediment HOC concentrations

A total of ca 400 mL sediment was pooled together from the three sub-samples and sent to ALS Scandinavia for analysis of PAH and PCB concentrations following the conditions and requirements of the European Standard SS EN 16167:2018 + AC2019 for PCB, and ISO/IEC 17025:2005 for PAH. The results are shown in Table 1.

Sediment geochemical characteristics

The three sub samples from all control cores were duplicated for the following analyses.

Sediment water content and loss on ignition (LOI):

Sediment was thawed and transferred to tared and pre-ignited crucibles and weighed before drying at 60°C for 24 h. The crucibles were weighed and the water content of the sediment was calculated before ignition in a muffle furnace at 550°C for 4 h. LOI was then calculated as the sediment weight after ignition subtracted from the sediment dry weight (DW).

Total phosphorus (Tot P) in sediment:

Sediment was dried at 90°C for 24 h and ground to a fine powder. The samples were analysed by the SWEDAC accredited chemical lab at DEEP, Stockholm University, by colorimetric analysis on a segmented flow nutrient analyser system (OI Analytical, Flow Solution IV, Xylem Inc., USA; method #319528) by determination of formed antimony phosphomolybdenum blue after ignition at 500°C and persulfate leaching.

Total organic carbon (TOC) in sediment:

Sediment was dried at 90°C for 24 h and ground to a fine powder. Ca 1 μg was transferred to silver capsules with a drop of ultra-pure deionized water. The capsules were placed in an desiccator containing 25 mL concentrated HCl and left to dissolve carbonates for <24 h according to Hedges & Stern.[10] The silver capsules were enclosed in tin capsules and analysed for TOC by the SWEDAC accredited chemical lab at DEEP, Stockholm University, using an elemental analyzer (Flash 2000, ThermoFischer Scientific., USA) at 950°C according to the manufacturer's operation manual P/N 31712052.

Total organic nitrogen (Tot N) in sediment:

The same method as for TOC, described above, but samples were not treated with HCl.

SECTION S4 – SEDIMENT CHARACTERISTICS AND CONTAMINANT CONCENTRATIONS

The Brunnsviken sediment was determined to be a silty mud with 91% water content and a 17% loss-on-ignition. Sedimentary total organic carbon was 10% of DW, total P was 0.1% of DW, and total N was 0.9% of DW. These data are coherent with previous reports.[2,11]

Table 3. Organic contaminants PAHs, categorized according to their molecular weight into three groups, and PCBs that were included in the study. The classification for PAHs has been developed by the Swedish Environmental Protection Agency (SEPA). Notice that the organic carbon-water partitioning coefficient (log K_{OC}) increases with the molecular weight. Concentrations in sediment: 2-4 cm sediment depth in the control (CTRL) cores of the present study; concentrations at 0-2 cm sediment depth at the same sampling location reported by Hjorth et al.[2]; SEPA class 5 corresponds to the worst sediment environmental quality criteria, i.e. worst classification threshold. All measured concentrations of PAHs and PCBs were above SEPA's sediment environmental quality criteria threshold. Log K_{OC} values were obtained from references [12] and [13] for PAHs and PCBs, respectively.

**Group**	**Log K_{OC}**	**Congener**	**Concentration in sediment (µg kg^{-1} DW)**		
			**Present study**	**Hjorth *et al.* [2]**	**NV class 5 threshold [14]**
PAH-L (light weight PAH)	4.26	Naphthalene	180		63
	4.40	Acenaphthene	80		33
	5.11	Acenaphtylene	32		
PAH-M (medium weight PAH)	4.71	Fluorene	90		35
	5.20	Phenanthrene	320	340	150
	5.75	Anthracene	190	200	45
	5.79	Fluoranthene	1100	1000	390
	5.82	Pyrene	980	910	380
PAH-H (heavy weight PAH)	6.55	Benzo(a)anthracene	510	450	180
	6.60	Chrysene	330	380	200
	6.88	Benzo(b)fluoranthene	780	820	440
	6.88	Benzo(k)fluoranthene	370	420	180
	6.68	Benzo(a)pyrene	640	620	240
	6.82	Dibenzo(ah)anthracene	180		79
	7.13	Benzo(ghi)perylene	810	790	400
	7.57	Indeno(123cd)pyrene	760	930	530
ΣPAH_{11}			6790	6900	2800

ΣPAH15			6758		4700
ΣPAH16			7300		
PCB	6.64	PCB28	0	3,3	1,3
	6.63	PCB52	0	11	1,9
	7.09	PCB101	23	28	5,5
	7.63	PCB118	22	20	3,6
	7.73	PCB138	17	34	9,1
	7.78	PCB153	24	30	7,9
	7.95	PCB180	10	18	4,9
ΣPCB7			96	140	34

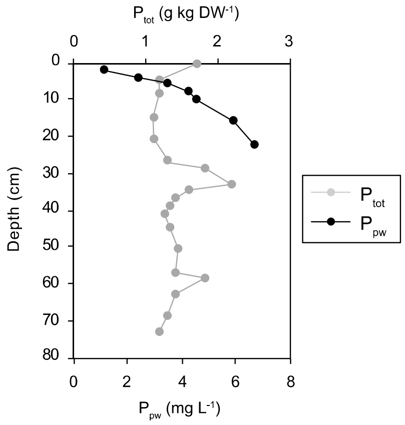

Figure 8. Sediment vertical profiles of concentrations of P in sediment (P_{tot}, grey) and porewater (P_{pw}, black). Data was collected at the same sampling location as in the present study, and kindly provided by Hjorth et al.[2].

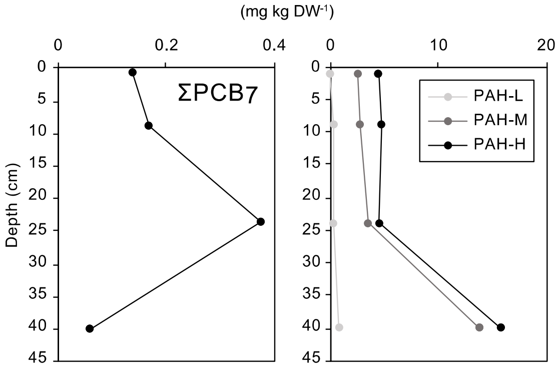

Figure 9. Sediment vertical profiles of concentrations of ∑PCB_7 and three weight-classes of PAH. Data was collected at the same sampling location as in the present study, and kindly provided by Hjorth et al.[2].

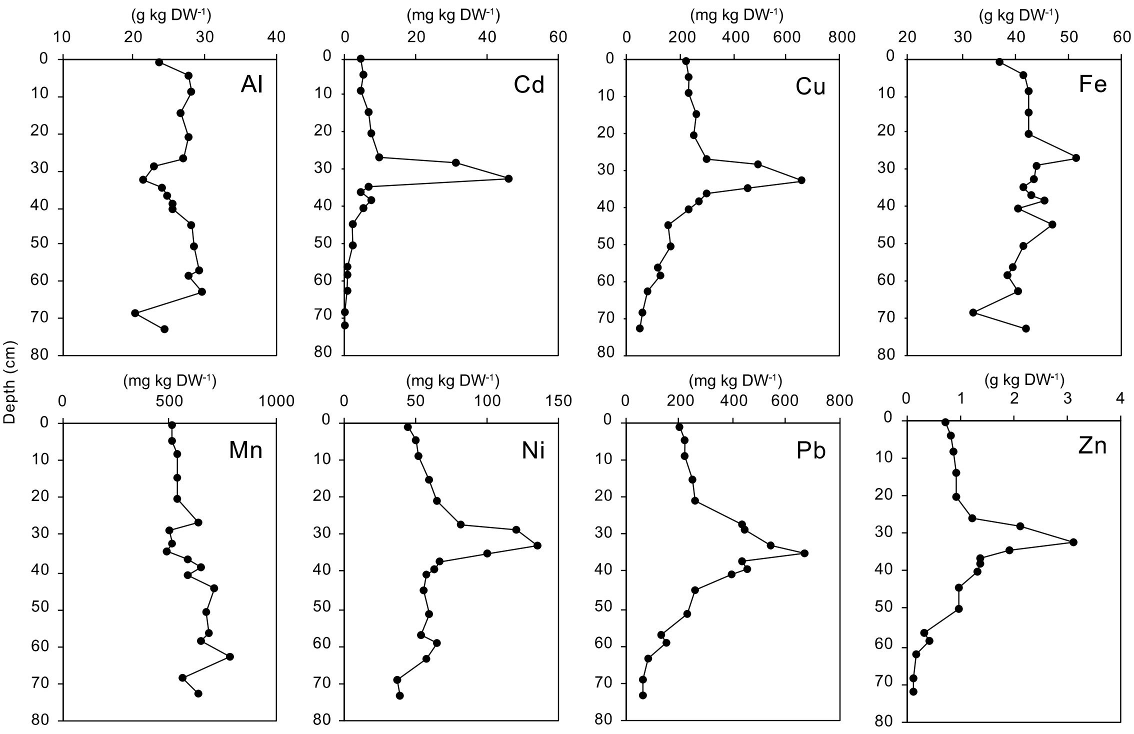

Figure 10. Sediment vertical profiles of concentrations of metals. Data was collected at the same sampling location as in the present study, and kindly provided by Hjorth et al.[2].

We used raw data kindly provided by Tomas Hjorth,[2] with the approval of Stockholm Municipality who ordered the data, to show the sediment vertical profiles over contaminants (Figure 7-9). In Hjorth et al., the sampling point is called B005 and is a good representative of an accumulating bottom in Brunnsviken. The sediment vertical profiles over metal concentrations show Al, Fe and Mn, that constitute a natural metal pool, have had little variation in the sediment accumulation during the last century (70 cm depth represent sediment from year ~1920). For the other, more toxic, Cd, Cu, Ni, Pb and Zn, a peak is seen at ~30 cm sediment depth, representing sediment deposited 1970.

SECTION S5 – DETAILS OF ANOVA AND PAIRWISE COMPARISONS

Table 2. Overview of measured parameters and corresponding statistical analyses. Differences between treatments ($p < 0.05$) are indicated by different letters for the treatments. If no significant difference was detected between treatments ($p > 0.05$), at least one letter is shared. CTRL = untreated control; AL = Al injected into sediment; AC = sediment capped with activated carbon, POL = sediment capped with Polonite.

Parameter	Analysis[a]		p-value	Differences between treatments				
Flux				CTRL	AL	AL+AC	POL	POL+AC
PO_4^{3-} (anoxic)	$F^*_{4,6.2}$	= 13.8	0.003	a	b	b	b	b
NH_4^+ (anoxic)	$F_{4,15}$	= 29.7	<0,001	a	b	b	a	a
NH_4^+ (oxic)	H_4	= 12.2	0.016	a	a, b	a	b	a, b
NO_X^- (oxic)	H_4	= 11.6	0.021	a	b	b	b	b
NO_X^- (anoxic)	H_4	= 1.9	0.752	-	-	-	-	-
ΣPCB_7	$F_{4,15}$	= 12.8	<0,001	a	b	c	a, b	c
ΣPAH_{16}	$F^*_{4,7}$	= 18.6	0,001	a	b	c	a	c
PAH-L	$F_{4,15}$	= 5.1	0,009	a	b	a	a	a

PAH-M	$F^*_{4,7.1}$	= 16.4	0,001	a	b	c	a	c
PAH-H	$F_{4,15}$	= 13.6	<0,001	a, b	a, c	c, d	b	d
O_2[b]	$F_{4,15}$	= 15.8	<0,001	a	b	b	a	b
O_2[c]	$F_{4,15}$	= 6.3	0,003	a	b	b	a, b	b
O_2[d]	$F_{4,15}$	= 42.5	<0,001	a	b	b	c	c
CH_4[b]	H_4	= 16.5	0,002	a	a, b	a, b	b	b
CH_4[c]	$F^*_{4,6}$	= 4.8	0,044	a	a	a	a	a
CH_4 (anoxic)[e]	$F^*_{4,6.6}$	= 14.1	0,002	a	a	a	b	b
DGT measurements								
Aluminum	$F^*_{4,6.8}$	= 5.33	0,029	a	b	a, b	a	a
Cadmium	$F^*_{4,6}$	= 18.2	0,002	a	b	a, b	b	a, b
Copper	H_4	= 12.8	0,013	a, b	a	a, b	b	a, b
Iron	H_4	= 13.3	0,010	a	b	a	a	a
Manganese	H_4	= 7.8	0,099	-	-	-	-	-
Nickel	$F^*_{4,7}$	= 11.5	0,003	a	a	b	a	a
Lead	H_4	= 14.4	0,006	a	b	a, b	a, b	a, b
PO_4^{3-}	H_4	= 7.8	0,013	a	a	a	a	a
Zinc	$F^*_{4,6.8}$	= 13.1	0,003	a	b	a, b	b	b
O_2 penetration depth	$F_{4,10}$	= 247.3	<0,001	a	b	c	d	d

[a]F = parametric with Student-Newmann-Keuls (SNK) post-hoc; F* = Welch's robust parametric for heteroscedastic data with SNK post-hoc; and H = non-parametric Kruskal-Wallis with Dunn's post-hoc. [b]Incubation 1. [c]Incubation 2. [d]Incubation 4. [e]Incubation 5.

SECTION S6 – WATER COLUMN PH

Table 3. Evolution of pH with time in all treatments during the experiment. pH values are mean ± standard deviation (n=4), and difference from control (CTRL). pH was measured in the water column above the sediment (using a pH meter Hach HQ40d) at the end of each incubation study. Values marked in bold were significantly different from the pH in the control sediment.

	**Incubation 1 (oxic)**		**Incubation 2 (oxic)**		**Incubation 3 (hypoxic) (passive sampling)**		**Incubation 5 (anoxic)**	
	Day 8		Day 22		Day 106		Day 135	
Treatment	pH ± sd	Difference	pH ± sd	Difference	pH ± sd	Difference	pH ± sd	Difference
CTRL	7.7 ± 0.12		7.5 ± 0.19		7.3 ± 0.11		6.9 ± 0.20	
AL	7.2 ± 0.14	**-0.5**	5.3 ± 0.14	**-2.2**	4.2 ± 0.17	**-3.1**	6.4 ± 0.06	**-0.5**
AL+AC	7.3 ± 0.08	**-0.4**	6.0 ± 0.57	**-1.5**	5.3 ± 0.82	**-2.0**	6.4 ± 0.04	**-0.5**
POL	9.1 ± 0.08	**+1.9**	8.4 ± 0.11	**+0.9**	7.5 ± 0.22	+0.2	7.4 ± 0.05	**+0.5**
POL+AC	9.0 ± 0.08	**+1.8**	8.1 ± 0.20	+0.6	7.7 ± 0.08	+0.4	7.3 ± 0.04	**+0.4**

SECTION S7 - EFFECTS OF ALUMINUM INJECTION ON METAL RELEASE IN SITU

Lake Växjösjön in southern Sweden was treated using Aluminum injection into the sediment in the summer of 2018.[15] In addition to sediment injection, Al was also added to the bottom water to precipitate P. This caused a drop in bottom water pH by ca 1 unit and a peak in Al concentrations at ca 200 µg L^{-1} in June in the small stream outlet at 0.5 m depth, according to public environmental monitoring data (Figure 11).[16] Al concentrations in the water column should be even lower if the solution is only added to the sediment. The pH effect was short-lived and confined to the treatment period of June to September. According to the same monitoring data, the Al treatment did not cause elevated water concentrations of Al, Pb, or any other toxic metals as we observed in the present study.

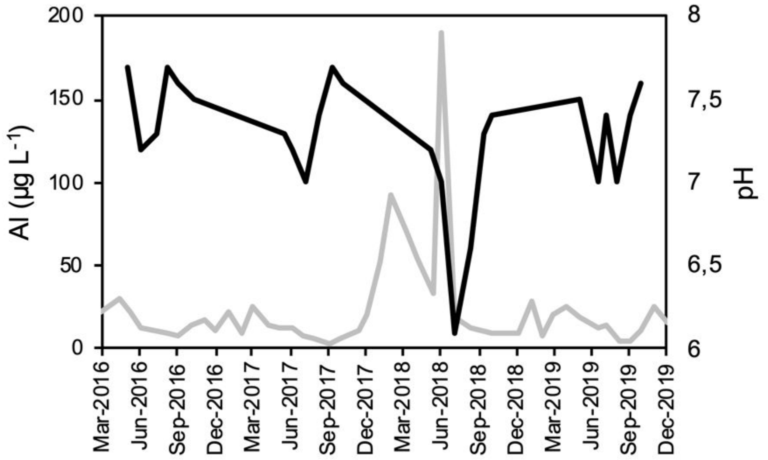

Figure 11. Time series from lake Växjösjön. Grey line shows Al concentration in the small stream outlet. Black line shows lake pH at 6m depth (bottom water).

REFERENCES

(1) Erlandsson, F. (personal communication July 2021). Stockholm Vatten Och Avfall (SVOA). Brunnsviken Monitoring Data. 2021.

(2) Hjorth, T.; Holmström, K.; Björinger, P. *Brunnsviken - Underlag För Lokalt Åtgärdsprogram – Sedimentprovtagning (Raw Data Provided by Stockholm Municipality and NIRAS 2021)*; 2017.

(3) Blomqvist, S.; Abrahamsson, B. An Improved Kajak-Type Gravity Core Sampler for Soft Bottom Sediments. *Swiss J. Hydrol.* **1985**, *47* (1), 81–84. https://doi.org/10.1007/BF02538187.

(4) Ramsing, N.; Gundersen, J. Seawater and Gases. Tabulated Physical Parameters of Interest to People Working with Microsensors in Marine Systems. *Unisense Online Resour.* **1994**, 20.

(5) Sithersingh, M. J.; Snow, N. H. Headspace-Gas Chromatography. In *Gas Chromatography*; Poole, C. F., Ed.; Elsevier Inc., 2012; pp 221–233.

(6) Wiesenburg, D. A.; Guinasso, N. L. Equilibrium Solubilities of Methane, Carbon Monoxide, and Hydrogen in Water and Sea Water. *J. Chem. Eng. Data* **1979**, *24* (4), 356–360. https://doi.org/10.1021/je60083a006.

(7) Davison, W.; Zhang, H. Introduction to DGT. In *Diffusive Gradients in Thin-Films for Environmental Measurements*; Davison, W., Zhang, H., Eds.; Cambridge University Press: Cambridge, 2016; pp 1–9. https://doi.org/10.1017/CBO9781316442654.002.
(8) Olofsson, R. S.; Rodushkin, I.; Axelsson, M. D. Performance Characteristics of a Tandem Spray Chamber Arrangement in Double Focusing Sector Field ICP-MS. *J. Anal. At. Spectrom.* **2000**, *15* (6), 727–729. https://doi.org/10.1039/b002609n.
(9) Rodushkin, I.; Ruth, T. Determination of Trace Metals in Estuarine and Sea-Water Reference Materials by High Resolution Inductively Coupled Plasma Mass Spectrometry. *J. Anal. At. Spectrom.* **1997**, *12* (10), 1181–1185. https://doi.org/10.1039/a702486j.
(10) Hedges, J. I.; Stern, J. H. Carbon and Nitrogen Determinations of Carbonate-Containing Solids1. *Limnol. Oceanogr.* **1984**, *29* (3), 657–663. https://doi.org/10.4319/lo.1984.29.3.0657.
(11) Rydin, E.; Jonsson, P.; Karlsson, M.; Gustafsson, A. *Läckagebenägen Fosfor i Brunnsvikens Sediment 2016. Naturvatten Rapport 2016:34*; 2016.
(12) Hawthorne, S. B.; Grabanski, C. B.; Miller, D. J. Measured Partitioning Coefficients for Parent and Alkyl Polycyclic Aromatic Hydrocarbons in 114 Historically Contaminated Sediments: Part 1. KOC Values. *Environ. Toxicol. Chem.* **2006**, *25* (11), 2901. https://doi.org/10.1897/06-115R.1.
(13) Hawthorne, S. B.; Grabanski, C. B.; Miller, D. J.; Arp, H. P. H. Improving Predictability of Sediment-Porewater Partitioning Models Using Trends Observed with PCB-Contaminated Field Sediments. *Environ. Sci. Technol.* **2011**, *45* (17), 7365–7371. https://doi.org/10.1021/es200802j.
(14) Josefsson, S. *Klassning Av Halter Av Organiska Föroreningar i Sediment*; 2017.
(15) Växjö Municipality. Bottenbehandling av Växjösjön och Södra Bergundasjön https://vaxjo.se/sidor/hallbar-utveckling/naturvard/sjoar-och-vattendrag/vaxjosjoarna/bottenbehandling.html (accessed Nov 6, 2020).
(16) Miljödata MVM http://miljodata.slu.se/mvm/Search (accessed Feb 17, 2020).
